# Morphological remodeling of *C. elegans* neurons during aging is modified by compromised protein homeostasis

**DOI:** 10.1038/npjamd.2016.1

**Published:** 2016-04-07

**Authors:** Elena M Vayndorf, Courtney Scerbak, Skyler Hunter, Jason R Neuswanger, Marton Toth, J Alex Parker, Christian Neri, Monica Driscoll, Barbara E Taylor

**Affiliations:** 1 Institute of Arctic Biology, University of Alaska Fairbanks, Fairbanks, AK, USA; 2 Department of Biology and Wildlife, University of Alaska Fairbanks, Fairbanks, AK, USA; 3 Warnell School of Forestry and Natural Resources, University of Georgia, Athens, GA, USA; 4 Department of Molecular Biology and Biochemistry, Nelson Biological Laboratories, Rutgers, The State University of New Jersey, Piscataway, NJ, USA; 5 Department of Neuroscience, CRCHUM, University of Montreal, Montreal, QC, Canada; 6 Laboratory of Neuronal Cell Biology and Pathology, Centre National de la Recherche Scientifique, Paris, France; 7 Sorbonnes Universités, UPMC Univ Paris 06, Paris, France

## Abstract

Understanding cellular outcomes, such as neuronal remodeling, that are common to both healthy and diseased aging brains is essential to the development of successful brain aging strategies. Here, we used *Caenorhabdits elegans* to investigate how the expression of proteotoxic triggers, such as polyglutamine (polyQ)-expanded huntingtin and silencing of proteostasis regulators, such as the ubiquitin–proteasome system (UPS) and protein clearance components, may impact the morphological remodeling of individual neurons as animals age. We examined the effects of disrupted proteostasis on the integrity of neuronal cytoarchitecture by imaging a transgenic *C. elegans* strain in which touch receptor neurons express the first 57 amino acids of the human huntingtin (*Htt*) gene with expanded polyQs (128Q) and by using neuron-targeted RNA interference in adult wild-type neurons to knockdown genes encoding proteins involved in proteostasis. We found that proteostatic challenges conferred by polyQ-expanded Htt and knockdown of specific genes involved in protein homeostasis can lead to morphological changes that are restricted to specific domains of specific neurons. The age-associated branching of PLM neurons is suppressed by N-ter polyQ-expanded Htt expression, whereas ALM neurons with polyQ-expanded Htt accumulate extended outgrowths and other soma abnormalities. Furthermore, knockdown of genes important for ubiquitin-mediated degradation, lysosomal function, and autophagy modulated these age-related morphological changes in otherwise normal neurons. Our results show that the expression of misfolded proteins in neurodegenerative disease such as Huntington’s disease modifies the morphological remodeling that is normally associated with neuronal aging. Our results also show that morphological remodeling of healthy neurons during aging can be regulated by the UPS and other proteostasis pathways. Collectively, our data highlight a model in which morphological remodeling during neuronal aging is strongly affected by disrupted proteostasis and expression of disease-associated, misfolded proteins such as human polyQ-Htt species.

## Introduction

The aging human brain displays remarkable morphological, physiological and behavioral plasticity.^1^ Whereas some individuals retain excellent cognitive function into their nineties and beyond, others succumb to devastating neurological decline as early as midlife. A common hallmark of brain aging is protein misfolding, a proteotoxic phenomenon that causes chronic cellular stress and is accompanied by protein aggregation.^2^ The progressive disruption of proteostasis is associated with a broad spectrum of neurodegenerative disorders such as Huntington’s disease (HD), Alzheimer’s disease and Parkinson’s disease.^3^ Brains impacted by these diseases can exhibit a series of morphological changes such as alteration of neuronal morphology and brain structure, which is believed to precede atrophy and degeneration of specific brain areas.^4^ Understanding early events that lead to brain atrophy and degeneration should shed light on neurodegenerative disease pathogenesis and facilitate design of effective interventions. HD, a dominantly inherited disease that is associated with expanded polyQ tracts (polyQs) in the huntingtin protein and neuronal dysfunction and death in the caudate nucleus and cortex, provides a tractable model in which to study these phenomena. The neurons of HD patients show abnormal dendritic growth, recurved distal dendritic segments and increased dendritic spine density,^5^ suggesting that morphological alteration is an important component of the pathogenic process in the disease.

We investigated morphological changes *in vivo* at the single-cell level using the *Caenorhabdits elegans* model. Similar to disease-free human neuronal aging, the naturally aging *C. elegans* nervous system undergoes striking changes in synaptic integrity and neuronal cytoarchitecture with age.^6–9^ Synapse-rich regions of the animal’s primitive brain (nerve ring) show a decrease in synaptic vesicle number, and an increase in vesicle-depleted synapses with the overall size of the presynaptic terminal reduced in cross-section.^7^ Several types of neurons (e.g., touch receptor, dopaminergic, GABAergic and cholinergic) show marked age-related sprouting from neuronal processes and somas. The mechanisms responsible for this restructuring are largely unknown. Moreover, aberrant neurons are also a hallmark of several *C. elegans* models of neurodegeneration,^10–13^ making the transparent and genetically malleable *C. elegans* a powerful model in which to investigate how individual neurons respond to proteotoxic challenge.^14^ In particular, *C. elegans* animals have six easily visualized touch receptor neurons, the anterior ALML, ALMR and AVM and posterior PLML, PLMR, and PVM.^15^ All six neurons exhibit morphological decline with age.^7^ ALM neurons control the response to gentle touch in the anterior body region whereas PLM neurons control gentle-touch response in the posterior region. Touch receptor neurons with over-expression of polyQ-expanded exon-1 human huntingtin exhibit progressive morphological abnormalities and loss of anterior and posterior touch response.^10,16,17^ These polyQ animals, therefore, provide a suitable model in which to investigate the morphological and functional response to proteotoxicity, at the single neuron level and in the course of aging.

Quality and integrity of the proteome is maintained by a tightly regulated network of molecular pathways and processes, including the ubiquitin–proteasome system (UPS), autophagy-mediated proteolysis, endoplasmic reticulum-associated degradation (ERAD), and the unfolded protein response (UPR). We hypothesized that disrupted protein homeostasis may induce the appearance of aberrant morphological phenotypes with age. Thus, neurons with abundant misfolded protein, and compromised proteostasis would be unable to clear misfolded and aggregated proteins, resulting in cellular dysfunction, functional decline, and morphological changes during aging. Given overwhelming evidence for proteostasis disruption in neuronal aging and neurodegenerative conditions,^3,18^ we tested the hypothesis that age-associated morphological changes in neurons result from disrupted proteostasis and that neurodegenerative proteins may impact morphological remodeling of aging neurons. To this end, we assessed the structural morphology of aging neurons in a proteostasis-challenged transgenic *C. elegans* strain in which touch receptor neurons express the first 57 amino acids of the human huntingtin gene with expanded polyQ repeats (128Q).^10,14,16^ In addition, we measured the function of wild-type and polyQ128 neurons, and tested the potential for functional correlation with morphological changes. Finally, we used neuron-targeted RNA interference (RNAi) in adult wild-type animals to knockdown genes involved in maintaining protein homeostasis followed by morphology scoring.

We report that proteostatic challenges conferred by polyQ-expanded huntingtin species and knockdown of specific genes involved in protein homeostasis can modify the frequency of morphological changes in aging *C. elegans* touch receptor neurons. We provide evidence of specific morphological changes such as extended outgrowths that are restricted to particular domains of touch neurons associated with compromised protein homeostasis. We also show that morphological remodeling of touch neurons during healthy aging can be regulated by the UPS and other protein clearance pathways. Our data highlight a model in which the dynamics of protein clearance and morphological remodeling during neuronal aging are strongly affected by disrupted proteostasis and expression of disease-associated, misfolded proteins such as polyQ-huntingtin.

## Results

### Proteostasis challenges conferred by expanded polyQ increase the frequency of soma outgrowth abnormalities in aging ALM neurons

In young adult *C. elegans*, touch receptor processes are straight except for major branches positioned near the nerve ring for ALM and AVM neurons; PLM neurons generate a synaptic branch that connects axons in the ventral cord. As animals age, wild-type touch receptor neurons accumulate striking morphological abnormalities.^7–9^ Previously, we and others have shown that subsets of aging wild-type touch receptor neurons exhibit distinctive aberrant structural features, with anterior ALMs prone to novel outgrowth from the soma, and PLMs prone to process branch abnormalities.^7–9^

To test the hypothesis that age-associated morphological abnormalities might result from disrupted proteostasis, we first characterized neurons expressing aggregation-prone human huntingtin proteins. We compared previously characterized morphological features^7^ of fluorescently labeled wild-type touch receptor neurons (*P*_*mec-4*_GFP) to fluorescently labeled touch receptor neurons that express the first exon of the human huntingtin gene linked to either the non-toxic polyQ19, or the toxic polyQ128 polyQ expansion protein.^10^ PolyQ128 animals accumulated aggregates with age (Supplementary Figure S1).

**Figure 1:**
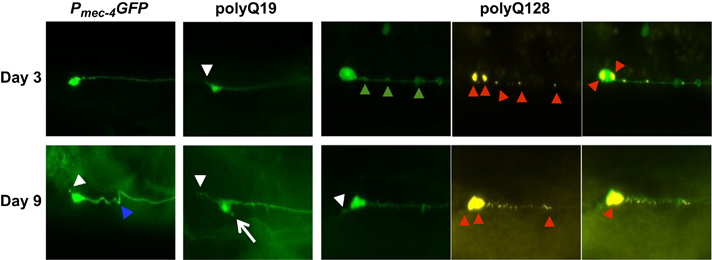
Representative images of morphological changes in touch receptor ALM neurons in *P*_*mec-4*_GFP, polyQ19 and polyQ128 animals at young and old ages. For polyQ touch receptor neurons, YFP-labeled neurons are in green, CFP-labeled polyQ128 huntingtin aggregates in yellow. Arrowheads and arrows denote neuronal aberrations (short soma outgrowth, white arrow; kinks, blue arrowhead; punctae, green arrowheads; polyQ aggregates, red arrowheads). All images were collected at ×20 magnification with constant exposure. (Imaged: *N*=150, 115 and 121 ALM neurons for *P*_*mec-4*_GFP, polyQ19 and polyQ128 strains, respectively, on day 3 of adulthood; *N*=107, 124 and 104 ALM neurons on day 9 of adulthood for *P*_*mec-4*_GFP, polyQ19 and polyQ128 strains, respectively). CFP, cyan fluorescent protein; GFP, green fluorescent protein; PolyQ, polyglutamine; YFP, yellow fluorescent protein.

**Figure 2:**
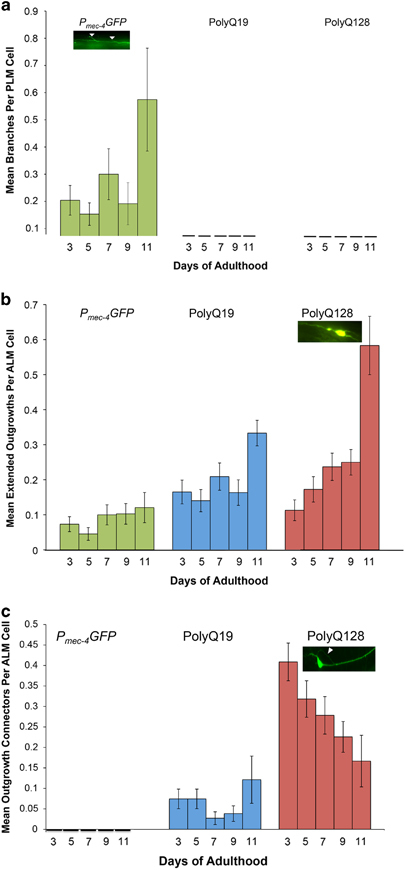
Neuronal aberrations in *P*_*mec-4*_GFP, polyQ19 and polyQ128 neurons across the lifespan. Animals were grown at 25 °C and imaged on the indicated days. Each bar represents mean±s.e. (**a**) Branches per PLM cell increased slightly in *P*_*mec-4*_GFP, but not in polyQ19 or polyQ128 animals (*P*_*mec-4*_GFP* N*=512, *P*=0.893; polyQ19 *N*=277, *P*=0.692; polyQ128 *N*=430; *P*=0.898; Wald test). (**b**) Extended outgrowths per ALM cell increased significantly in polyQ128, but not in *P*_*mec-4*_GFP or polyQ19 animals (*P*_*mec-4*_GFP* N*=557, *P*=0.257; polyQ19 *N*=489, *P*=0.126; polyQ128 *N*=482, *P*=0.009). (**c**) There were significantly more outgrowth connectors per ALM cell in polyQ128 animals on day 3 (*P*<0.00001) compared with polyQ19 and *P*_*mec-4*_GFP. A numerical decrease in these connectors over time was not statistically significant (*P*=0.213) (*P*_*mec-4*_GFP* N*=557; polyQ19 *N*=489; polyQ128 *N*=482). GFP, green fluorescent protein; PolyQ, polyglutamine.

Neurons of animals with expanded, toxic polyQ repeats (polyQ128) accumulated morphological abnormalities with age, a phenomenon also observed in aging touch neurons without expressed toxic proteins (Figure 1). We found that specific abnormality types differed considerably among strains and cell types. Branches increased over time in *P*_*mec-4*_GFP PLM neurons, consistent with previous findings.^7^ However, neither polyQ19 nor polyQ128 PLM neurons exhibited significant branching with age (Figure 2a, Supplementary Figure S3a).

Extended outgrowths from the soma of ALM neurons became noticeable around day 5 of adulthood and increased thereafter, to an extent that varied by strain. PolyQ128 neurons exhibited an increase in extended outgrowths with age (Wald test, *P*=0.009) and also had significantly more outgrowths with age compared to *P*_*mec-4*_GFP and polyQ19 animals (Figure 2b, Supplementary Figure S3b). Statistically significant differences were evident on day 9 of adulthood and persisted until the end of life. Thus, polyQ expansion was linked to the production of long outgrowths specifically from the ALM soma.

We designated another soma abnormality subtype, specific to polyQ ALM neurons, as ‘outgrowth connectors’—outgrowths extending from the cell body that loop back and connect to the soma or process (Figure 2c, Supplementary Figure S3c). In polyQ19 and polyQ128 lines, outgrowth connectors were present at first imaging on day 3. We detected outgrowth connectors in polyQ19 (mean over all days=0.06/neuron), and many more in polyQ128 (mean over all days=0.30/neuron; Wald test for differences between polyQ19 and polyQ128 on day 3, *P*<0.0001). However, prevalence of outgrowth connectors did not change significantly over time, although there was a slight trend toward decline with age in polyQ128 (Wald test, *P*=0.21). We found no outgrowth connectors in *P*_*mec-4*_GFP neurons. Thus, outgrowth connectors appear to be persistent from early adulthood onward and associated with increased polyQ load.

### Charcterization of neuronal dysfunction

**Figure 3:**
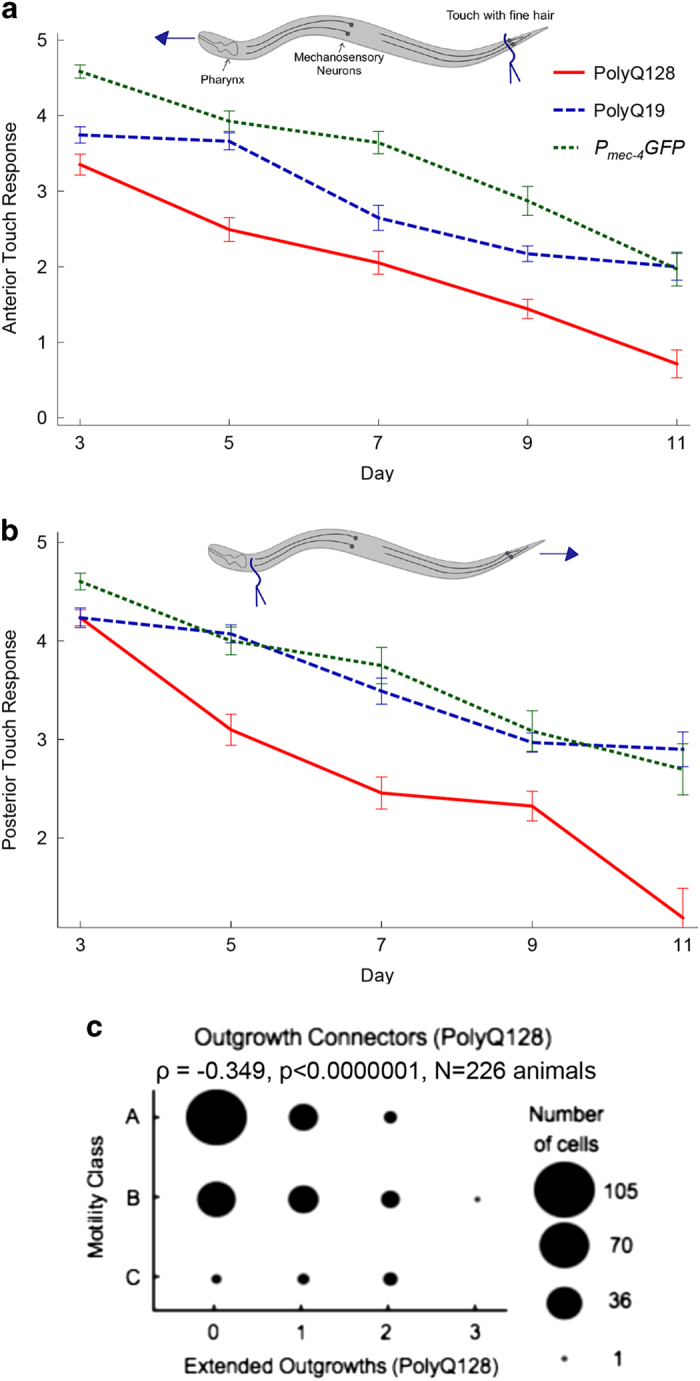
Gentle touch response across the lifespan in *P*_*mec-4*_GFP, polyQ19 and polyQ128 animals. Animals were grown at 25 °C and tested for gentle-touch response on the indicated days. Anterior (**a**) and posterior (**b**) gentle-touch response during adulthood in *P*_*mec-4*_*GFP*, polyQ19 and poly128 animals measured on a scale of number of responses to 5 tests of gently provoking animals on the tail or head with an eyelash. The analysis was run on three independent trials and a compilation of observations per time point is presented to obtain each N. Each bar represents mean±s.e. There was a statistically significant decrease in both anterior and posterior touch response in every strain (*N*=*P*<0.0001, Wald test). No differences in slope were detected among strains with the exception of anterior touch response polyQ19 versus polyQ0 (*P*=0.02). Anterior: polyQ0 *N*=65 (d3), 68 (d5), 56 (d7), 47 (d9), 33 (day11); polyQ19 *N*=51 (d3), 56 (d5), 51 (d7), 64 (d9), 33(d11); polyQ128 *N*=68 (d3), 61 (d5), 57 (d7), 68 (d9), 21 (d11). Posterior: polyQ0 *N*=73 (d3), 71 (d5), 56 (d7), 47 (d9), 33 d11); polyQ19 *N*=51 (d3), 56 (d5), 51 (d7), 64 (d9), 20 (d11); polyQ128 *N*=68 (d3), 61 (d5), 57 (d7), 68 (d9), 21 (d11). (**c**) Spearman correlation plot: extended outgrowths in polyQ128 animals versus mobility. Youthful animals move spontaneously (Class A) and progressively decline as they age, requiring gentle prodding to move (Class B), or barely move at all even after prodding (Class C).^20^ (*N*=226, *ρ*=−0.349, *P*<0.001). d, day; GFP, green fluorescent protein; PolyQ, polyglutamine.

Whether specific morphological changes in aging touch neurons are associated with neuronal impairment is not clear.^7–9^ To address whether form reflects function in the polyQ model, we first established a pattern of functional decline in our model strains (Figures 3a,b). Next, we probed for correlations between morphological abnormalities and function, with cell-type-specific analysis. For naturally aging PLM neurons in the *P*_*mec-4*_GFP line, branches did not correlate with compromised posterior touch response over adult life (*ρ*=0.07, *P*=0.28). The lack of branches in PLM neurons of polyQ19 or polyQ128 animals precluded correlation analyses for these strains. Thus, branches did not predict posterior touch dysfunction for aging wild-type touch neurons, consistent with recent suggestions that branches might actually be preferentially produced in successfully aging PLM neurons.^19^

Branched ALM neurons in the *P*_*mec-4*_GFP strain, a rare age-associated abnormality for this cell type,^7^ showed a weak but significant inverse correlation with anterior touch response, suggesting that branches in ALM neurons may be associated with different biological outcomes (*ρ*=−0.134, *P*=0.043, *N*=228 animals). Occasional branches were present in ALM neurons of polyQ128 animals, but their presence did not correlate with anterior touch response (*ρ*=0.009, *P*=0.90, *N*=216 animals).

Aging is often accompanied by a decline in physical and physiological health parameters such as muscle size and strength, mobility and metabolism. We assessed whether the presence of branches, extended outgrowths or outgrowth connectors was associated with the animals’ mobility, which is one measure of healthspan commonly used in aging studies.^20^ Among age-synchronized polyQ128 animals, the presence of extended outgrowths was strongly inversely correlated with mobility (*ρ*=−0.349, *P*<0.0001, *N*=226 animals; Figure 3c). These outgrowths were not common enough in polyQ19 and *P*_*mec-4*_GFP animals to warrant analysis. Neither branches nor outgrowth connectors were correlated with reduced mobility in any cell type or strain.

### RNAi knockdown of proteostasis components can affect neuronal morphology and function of wild-type touch receptor neurons

We selected 26 candidate genes impacting various facets of protein homeostasis through RNAi, including chaperones, ER stress/UPR components, ERAD degradation proteins, proteins involved in ubiquitin-mediated degradation, kinases and other genes previously reported in the literature to suppress or enhance polyQ or other protein aggregation toxicity, disaggregation and/or protein toxicity (see Supplementary Tables S1 and S2).^17,21–40^ Using an RNAi approach in a strain sensitized for neuronal RNAi and expressing green fluorescent protein (GFP) in the touch neurons, we knocked down each gene starting at developmental stage late L4 and assessed morphological and functional end points on adult day 5. We emphasize that our approach features expression knockdown only in wild-type adult animals sensitized to pan-neuronal RNAi, so outcomes do not address developmental consequences of disruption.

#### Proteasome

**Figure 4:**
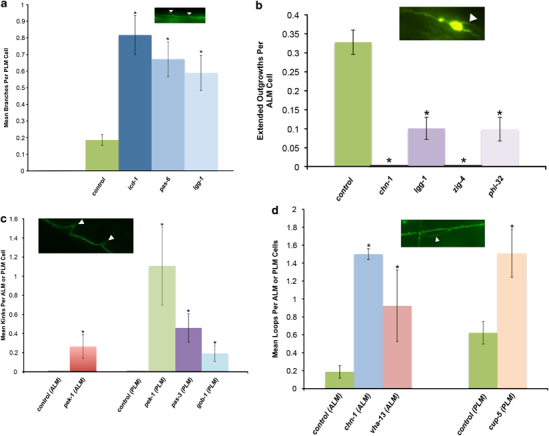
Effects of neuron-targeted RNAi knockdown of proteostasis components on the neuronal morphology of naturally aged touch receptor neurons. *P*_*mec-4*_GFP animals were treated with RNAi for the corresponding gene starting at the late L4 stage and morphological changes, either branches (**a**) extended outgrowths (**b**), kinks (**c**) or loops (**d**) were quantified on day 5 of adulthood. Data were analyzed using the Mann–Whitney *U*-test with Holm–Sidak step-down comparisons. Branches: *N*=120, 131, 107, 242 neurons for *icd-1, pas-6, lgg-1* and empty vector control, respectively. Branches: *P*⩽0.004 for each gene versus the corresponding control. Extended outgrowths: *N*=96, 109, 38, 91, 268 neurons for *chn-1, lgg-1, zig-4, phi-32* and empty vector control, respectively. Extended outgrowths: *P*⩽0.011 for each gene versus the corresponding control. Kinks: *N*=52, 48, 53, 56 and 172 neurons for *gob-1*, *pas-3*, *pek-1* (ALM), *pek-1*(PLM) and empty vector control, respectively. Kinks: *P*<0.01 for each gene versus the corresponding control. Loops: *N*=96, 26, 55 and ⩾172 for *chn-1*(ALM), *vha-13*(ALM), *cup-5*(PLM) and empty vector control, respectively. Loops: *P*<0.04 for each gene versus the corresponding control. GFP, green fluorescent protein; RNAi, RNA interference.

We knocked down four genes that make up subunits of the proteasome: *pas-3*, an alpha type 4 subunit of the 20S core particle; *pas-6*, a type 1 alpha subunit of the 20S core particle; *rpt-5*, a triple A ATPase of the 19S regulatory particle, and *aip-1*, a negative regulatory particle of the 19S proteasome (*aip-1 *RNAi activates the proteasome and can be neuroprotective against polyQ128). Disruption of two of the four genes increased aberrant PLM morphology: *pas*-*6* increased branches by 261% from a mean of 0.186 to 0.672 aberrations/cell (Table 1, Figure 4a), *pas-3* increased kinks from 0 to 0.458 kinks/cell (Table 2, Figure 4c), and *rpt-5* and *aip-1* had no significant effect (Supplementary Tables S1 and S2). Our data suggest that the integrity of the core 20S subunit of the proteasome may have an important role in the maintenance of PLM process morphology.

### Lysosomal function

We tested three lysosomal genes: *cup-5*, *lmp-1* and *vha-13,* for impact on morphology of aging touch neurons. *Cup-5* RNAi produced PLM neurons with a 143% increase in loops, from a mean of 0.622 to 1.509 loops/cell, as compared to the empty vector control (Table 2, Figure 4d). The RNAi of *vha-13*, a lysosomal ATPase, increased loop formation by 394% from 0.187 mean occurrences per cell in empty-vector-control-treated neurons to 0.923 in ALM processes (Table 2, Figure 4d). *Lmp-1(RNAi)*, a lysosomal-associated membrane protein had no effect on neuronal morphology (Supplementary Tables S1 and S2). Thus, our results suggest that disrupting lysosomal function can lead to loop formation in neuronal processes.

### Ubiquitin pathway

We knocked down five genes active in the ubiquitin pathway: *phi-32* (ubiquitin-like protein), *let-70* (ubiquitin-conjugating enzyme), *chn-1* (ubiquitin ligase),* ufd-1* (ubiquitin fusion degradation protein), and *cdc-48.1* (ubiquitin-selective chaperone). Of these interventions, *chn-1* (RNAi) and *phi-32* (RNAi) affected neuronal morphology by decreasing the number of extended outgrowths in ALM neurons from 0.328 in empty-vector-control-treated neurons to 0 in *chn-1* RNAi-treated neurons, and 0.099 in *phi-32* RNAi treated neurons (Table 1, Figure 4b). *Phi-32* was one of only two genes (the other being B0041.5, posterior only) whose knockdown also significantly reduced the animals’ anterior and posterior touch response. *Chn-1* knockdown also increased ALM loops from 0.187 to 1.5 mean occurrences per cell (802%) (Table 2, Figure 4d) suggesting modulation of multiple phenotypes. Interestingly, both *chn-1* and *phi-32* exclusively affected ALM, but not PLM neurons.

### Autophagy

We performed RNAi on two genes that promote macroautophagy, *lgg-1* and *bec-1*. *Lgg-1* (RNAi) increased PLM branching from 0.186 mean occurrences in empty vector control to 0.589 in treated neurons (217%) and decreased extended outgrowths in ALMs by 69% (from 0.328 to 0.101 outgrowths/cell) (Table 1, Figures 4a,b). We also observed a reduced anterior and posterior touch response, although it did not quite reach the level of significance (*P*=0.06). *Bec-1* (RNAi), which targets expression of a protein that localizes autophagy proteins to pre-autophagosomal structures, had no effect (Supplementary Tables S1 and S2).

### ER stress/UPR

In mammals, the UPR can be activated through 3 distinct pathways: IRE-1/XBP-1, PERK and ATF-6. We tested two *C. elegans* UPR protein homologs: *ire-1* *and pek-1*. We also knocked down *icd-1*, a subunit of the nascent-associated complex that can induce ER stress when it is disrupted. RNAi of *pek-1* and *icd-1* significantly increased the accumulation of kinks (0 to 0.264 kinks in ALM and 0 to 1.107 kinks in PLM neurons for *pek-1*; Table 2, Figure 4c) and branches (0.186 to 0.817 branches in PLM neurons with *icd-1* or 339%; Table 1, Figure 4a). Knockdown of *ire-1* had no detectable effect (Supplementary Tables S1 and S2). RNAi of *icd-1* increased the number of PLM branches by 339% (Table 1, Figure 4a), whereas RNAi of *pek-1* increased kinks in both ALM (from 0 to 0.264 mean kinks/cell), and PLM neurons (from 0 to 1.101 mean kinks/cell; Table 2, Figure 4c). These observations suggest that disrupting the PEK-1 pathway of the UPR leads to specific and distinct morphological changes in touch neurons.

## Discussion

Our study highlights two important aspects of the relationship between proteostasis and neuronal aging. First, our data suggest that the regulation of proteostasis by protein clearance pathways such as the UPS has a major influence on morphological remodeling of naturally aged neurons. We find that most pathways tested are not tied to a particular morphological outcome, but rather are associated with several neuronal aberrations suggestive of the complexity of proteostasis subpathway interactions. Second, proteostress induced by neurodegenerative disease proteins such as polyQ-Htt markedly alters neuronal aging at the morphological level by increasing the frequency of specific neuronal aberrations from the soma. Our findings suggest that proteostress, induced both internally, in the form of expanded polyQ expressed in the neurons, and externally, as with disruption of protein homeostasis through pan-neuronal RNAi, modifies morphological remodeling of aged neurons. Overall, our work extends previous reports that showed that some classes of *C. elegans* neurons accumulate abnormal morphologies and lose function with age, and that these changes can be modulated by components of the proteostasis-modulating insulin signaling pathway, the transcription factor *hsf-1*, and *jnk-1*.^7–9^

### Distinct structural outcomes are a hallmark of aggregation-prone polyQ128 ALM and PLM neurons

Several *C. elegans* models of neurodegeneration have shown that protein aggregation with age can lead to aberrant neuronal form and function.^11–13^ Faber *et al.* ^11^ showed that expressing the human huntingtin gene with 150 polyQs caused *C. elegans* ASH sensory neurons to adopt a speckled bag morphology, swell to two to three times their normal size and in some cases lose all intracellular content. The animals also exhibited a severe defect in the nose touch response. Similarly, Miyasaka and colleagues^12^ showed that expressing the human mutated tau gene in touch receptor neurons reduced touch sensitivity, increased neuronal abnormalities, and caused the loss of microtubular spindles. By day 5 of adulthood, ALM neurons showed multiple outgrowths and branches, and by day 10 these neurons were dead.

Similar to the tauopathy models, we found that anterior and posterior neurons with polyQ-expanded Htt expression had distinct neuronal morphologies. Anterior touch neurons in polyQ128 animals had significantly more morphological abnormalities associated with the soma of touch receptor neurons; posterior neurons trended toward more aberrations in the processes, which consisted mainly of branched processes. We observed fewer branches in PLM processes and more outgrowths in ALM processes of polyQ-expressing neurons compared to aging wild-type touch receptor neurons.

### Relationship between function and morphological remodeling of aging touch neurons

Our analysis detected one significant inverse relationship, between branches in wild-type ALM neurons and anterior touch response. Overall, we did not observe strong sensory incapacity as a consequence of morphological abnormalities. However, whether individual neuronal aberrations result in circuit disruption remains to be investigated. It is likely that subtle and progressive differences in neuronal response occur somewhat coincident with, or related to, morphological change but not directly tied to it. Moreover, we previously showed that a morphological abnormality (PLM branching) can be associated with improved function (posterior touch response)^19^ raising the possibility that compensatory mechanisms may be involved. The relationship between neuronal morphology and function might best be resolved by direct electrophysiological characterization of the health of individual neurons or by use of gene-based indicators of neuronal activity such as the genetically encoded calcium indicator, GCaMP. Such studies would likely need to test many neurons to detect functional differences.

Interestingly, we also observed an inverse relationship between mobility and extended outgrowths from the soma. In this regard, it is unlikely that extended outgrowths *per se* impair mobility. Rather, we suggest that these aberrations are symptomatic of changes within the neuron that correlate with functional problems. From this perspective, a neuron with a soma outgrowth is exhibiting signs of the aged phenotype, and therefore, might be more likely to succumb to accelerated decline and dysfunction.

### The UPS pathway is a major regulator of morphological remodeling of middle-aged wild-type touch neurons

**Figure 5:**
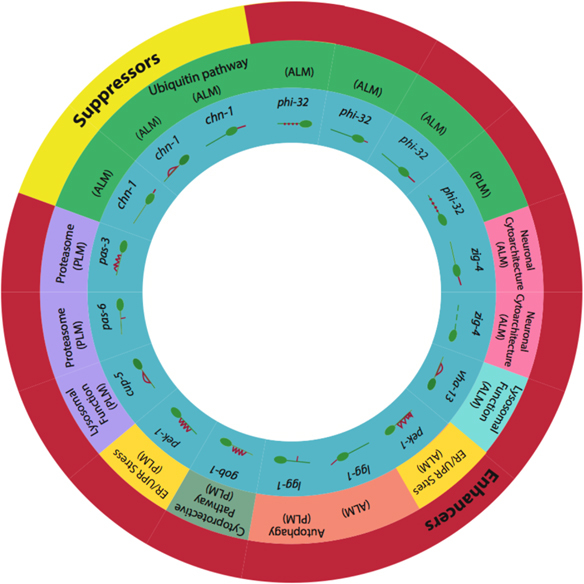
Summary of significant results from RNAi proteostasis screen. Late L4 developmental stage *P*_*mec-4*_GFP animals (strain ZB4064) were cultured at 25 ° from hatching, treated with neuron-targeted RNAi at the late L4 stage, and assessed for neuronal morphology integrity on day 5 of adulthood. Enhancers or suppressors in outer ring refer to previously published reports of enhancement or suppression of polyQ or other protein aggregation toxicity, disaggregation and/or protein toxicity.^17,21–40^ GFP, green fluorescent protein; RNAi, RNA interference.

Of the genes we tested, those in the UPS pathway were the most likely to produce a morphological effect when knocked down. The UPS was also the only pathway that induced all four of the major types of morphological changes (branches, extended outgrowths, kinks and loops) when individual components were knocked down. Knockdown of four genes from the UPS pathway significantly affected neuronal structure: *phi-32* (ubiquitin-like protein), *chn-1* (E3 ubiquitin ligase), *pas-6* (proteasome subunit), *pas-3* (proteasome subunit) (Tables 1 and 2, Figure 5). The identification of two genes that code for the catalytic 20S particle of the 26S proteasome, highlights this molecular degradation engine as important for the maintenance of neuronal morphology in aging touch neurons.

We also tested five genes for which RNAi was previously reported to suppress polyQ toxicity. We found that disrupting genes whose RNAi was formerly shown to promote proteostasis (enhancers) was much more likely to result in a neuronal aging phenotype (19 out of 22 genes) compared to genes whose RNAi suppresses proteostasis (1 out of 5). The E3 ubiquitin ligase *chn-1* was the sole suppressor to affect neuronal morphology in our RNAi screen. Interestingly, in contrast to polyQ PLM neurons (which are generally suppressed for age-associated branches), *pas-6* RNAi increased PLM branching in naturally aged neurons. We speculate that the baseline level of stress and misfolded protein overload is likely to be significantly less under conditions of RNAi knockdown in a native background as compared to polyQ transgenic neurons. Partial knockdown of the proteasome via RNAi may contribute to mild, but not severe stress and lead to hormesis compensation, whereas the higher level of polyQ overload and stress might cross a toxicity threshold. In future studies, it would be of interest to compare the morphological and functional effects of knocking down the same proteoastasis-regulating genes in the neurons of both wild-type and polyQ animals, and the effects on polyQ aggregation in the polyQ animals. Overall, our findings suggest that misfolded protein clearance and turnover by the UPS are important in maintaining the integrity of neuronal structure, and that perturbing multiple steps in these processes may disrupt morphology of middle-aged touch receptor neurons.

### Disruption of autophagy, ER-associated UPR and lysosomal degradation in wild-type touch middle-aged neurons is associated with morphological remodeling

Our findings also implicate autophagy disruption in altered neuronal morphology. Of the two selected autophagy genes we tested, *bec-1* and *lgg-1*, only *lgg-1* (RNAi) produced a significant change in wild-type neuronal morphology. RNAi of *lgg-1* produced both a decrease in extended outgrowths and an increase in branches, only one of two genes in our screen to affect more than one phenotype. *Lgg-1* functions in phagophore elongation and autophagosome vacuole formation. In late-onset disorders such as Huntington’s, Alzheimer’s, ALS, and familial Parkinson’s disease, defects in the autophagy pathway are common. Interestingly, the autophagosomes of Huntington’s disease patients form normally and are cleared despite slower than normal macroautophagic protein turnover, suggesting that autophagosome membranes do not target substrates properly during sequestration.^41^

Two of three genes involved in the ER-associated unfolded protein response, *pek-1* and *icd-1*, affected neuronal morphology of wild-type neurons. We note that of two transmembrane proteins responsible for initiating the UPR cascade (three exist, our study tested two), only *pek-1* had an effect on morphology. Adaptive responses of *pek-1* activation include antioxidant protection, metabolism, and autophagy.^42^ Disrupting one or more of these processes may accelerate neuronal aging. RNAi of *ire-1*, a protein that initiates a parallel UPR pathway, failed to have an effect on neuronal morphology under our experimental conditions, suggesting that some UPR pathways may be more critical than others. The effects of ERAD dysregulation on neuronal aging have been studied in yeast cells and neuron-like PC12 cells expressing polyQ-expanded huntingtin fragments. Impaired ER protein homeostasis is known to contribute to polyQ toxicity in yeast, PC12 cells, and striatal cells expressing full-length polyQ-expanded huntingtin.^43^

Alterations in lysosomal degradation occur in both normal brain aging and in age-related neurodegenerative diseases. In our screen, RNAi of genes that regulate lysosomal function affected neuronal morphology. Interestingly, of the lysosomal genes we tested, *cup-5* and *vha-13* always produced looping of either ALM or PLM processes, suggesting that compromised lysosomal degradation affects the integrity of healthy, smooth processes.

### Overall conclusions from disruption of protein homeostasis by neuronal RNAi and polyQ-expanded Htt species

We tested the hypothesis that proteotoxic stress contributes to aberrant neuronal morphology by disrupting selected genes that both negatively and positively regulate the UPS, ERAD, autophagy and lysosomal pathways, as well as genes previously shown to be cytoprotective. Clones were selected for previous impact on polyQ, yet some (24%) of these did not change the ‘normal’ aging trajectory of day 5 middle-aged GFP labeled wild-type neurons, suggesting that different gene sets are important for different stresses. We emphasize that more than 75% of mostly proteostasis-related genes we examined induced abnormal morphology of wild-type neurons, supporting the hypothesis that proteostasis is an important factor in maintaining neuronal structure later in life (Figure 5). Disrupting individual genes within a single pathway sometimes, but not always, produced similar aberrations; thus, we cannot exclusively assign any given morphology to any specific proteostasis subpathway. Proteostasis is a complex process in which changing one component is likely to affect multiple pathways. Because it is unclear how a single gene intervention can reset efficiency of each proteostasis pathway, the finding that some seemingly oppositely directed perturbations induce similar outcomes is not entirely surprising. The poorly understood complexity of proteostasis subpathway interactions limits our ability to predict what a given RNAi intervention will do at the physiological level. This may explain cases in which both a neuroprotective and a neurocompromising change induced a similar cellular response. We also note that RNAi is not always equally effective for all genes; thus, negative results cannot be interpreted as a lack of an effect relative to the true biology of the model.

In summary, we propose that age-associated, neuronal morphological changes are the result of protein homeostasis dysregulation. As morphological remodeling of neurons during aging can be easily monitored in *C. elegans*, our data support that *C. elegans* models of neurodegenerative diseases constitute plausible models in which to search for genes, pathways, and drugs that promote or restore normal aging of diseased neurons. Overall, similarities in neuronal morphology and molecular pathways between *C. elegans* and humans could shed light on the role of neuronal proteostasis in age-associated neurological diseases, and spur the development of therapeutic interventions.

## Materials and methods

### *C. elegans* culture and maintenance

We used standard methods for strain maintenance, bacterial culturing, and animal manipulation.^44^ Animals were maintained on nematode growth medium (NGM) seeded with live *Escherichia coli* bacteria strain OP50-1. All experiments were conducted at 25 °C.

### Strains and crosses

We crossed a polyQ disease model (polyQ128): ID1 *igIs1[P*_*mec-7*_*yfp, P*_*mec-3*_*htt57Q128::cfp, lin-15(+)]* and corresponding polyQ control (polyQ19): ID245 *igIs245[P*_*mec-7*_*yfp, P*_*mec-3*_*htt57Q19::cfp, lin-15(+)],* which have yellow fluorescent protein-labeled touch receptor neurons and express the first 57 amino acids of the human huntingtin gene containing either 128 or 19 polyQ repeats tagged with cyan fluorescent protein (CFP)^10,14,16^ into TU2370 *uIs57[P*_*unc-119*_*SID-1, P*_*unc-119*_*yfp, P*_*mec-6*_*mec-6]*, a strain that pan-neuronally overexpresses the transmembrane channel SID-1 and allows targeting small interfering RNAs into the neurons.^45^ These crosses generated strains and genotypes: ZB4062 *igIs1[P*_*mec-7*_*yfp, P*_*mec-3*_*htt57Q128::cfp, lin-15(+)*]; *uIs57[P*_*unc-119*_*SID-1, P*_*unc-119*_*yfp, P*_*mec-6*_*mec-6]* and ZB4063 *igIs245[P*_*mec-7*_*yfp, P*_*mec-3*_*htt57Q19::cfp, lin-15(+)*]; *uIs57[P*_*unc-119*_*SID-1, P*_*unc-119*_*yfp, P*_*mec-6*_*mec-6]*. Animals expressing human huntingtin with either 19 or 128 glutamines show normal touch neuron morphology and response as larvae; defects do not emerge until the late L4 stage prior to young adulthood. In addition, we crossed *P*_*mec-4*_GFP*, lin-15(+)*, which labels wild-type touch receptor neurons with GFP, into TU2370 to generate strain ZB4064 *zdIs5[P*_*mec-4*_GFP*, lin-15(+)]; uIs57 [P*_*unc-119*_*SID-1, P*_*unc-119*_*yfp, P*_*mec-6*_*mec-6].* This latter strain was used as a non-polyQ control.

### Gentle touch response assays

Assays were performed based on the methods described by Calixto and colleagues.^45^ Briefly, we measured the touch response of 20 animals per group on a scale of number of responses to 5 tests of gently provoking animals with the touch of an eyelash on the tail or head. The assay was run in 3 independent trials.

### Neuronal morphology imaging

Following touch response assays on days 3, 5, 7, 9 and 11 of adulthood, we mounted animals onto cover slips using 2 μl of 36% w/v Pluronic™ solution in water and imaged using the ×20 objective of a Zeiss AxioVert S100 inverted fluorescence phase contrast microscope (Carl Zeiss, Jena, Germany). Using constant microscope settings, we collected images of the touch neurons of each individual and associated huntingtin protein aggregates, if present, using fluorescein isothiocyanate and CFP filters, respectively. Animals were discarded after imaging.

### Neuronal morphology assay and protein aggregate quantification

We imaged touch receptor neurons to quantify morphological abnormalities using the method of Toth *et al*.^7^ Aggregates were quantified using an ImageJ^46^ macro, which (1) selects the brightest points in an image (2) expands selections to a 15-pixel radius (3) uses a threshold pixel brightness value of at least 110 (out of 255 possible in 8-bit grayscale images) to delineate portions of the aggregate and (4) counts the number of detected aggregates larger than 4 pixels.

### Proteostasis RNA interference screen in wild-type neurons

To test the effect of knocking down candidate genes on touch receptor neuron morphology and function, we fed late L4 stage *P*_*mec-4*_GFP*; P*_*unc-119*_*SID-1, P*_*unc-119*_*yfp, P*_*mec-6*_*mec-6* (strain ZB4064) animals RNAi clones of *E. coli* strain *HT115* expressing the dsRNA of the target gene from the Ahringer Lab RNAi library. We applied RNAi treatments to populations synchronized using the egg-lay method. We placed 15 gravid adults on a seeded NGM plate to lay eggs for 4 h, and allowed their progeny to hatch. We transferred animals daily to ensure an age-matched population. At day 5 of adulthood, we tested animals for gentle-touch response and changes in neuronal morphology. For each batch of plates, we confirmed RNAi efficacy by knocking down GFP in the nerve ring of ZB4064 animals and comparing fluorescence to animals of the same strain who were fed the L4440 empty vector control. Only batches of plates that showed a significant (unpaired *t-*test, *P*-value<0.01) knockdown of GFP were used for further RNAi experiments (Supplementary Figure S2). Following an initial RNAi screen, we retested candidates two additional times. Results are reported from all trials combined.

### Statistical analysis

To analyze neuronal morphology, protein aggregates and gentle-touch response as functions of age and strain, we used generalized linear models with a logit link function (i.e. Poisson regression) for count data, and a log link function (i.e. logistic regression) for both binary data such as presence/absence of aberrations and touch response (which was rescaled to range from 0 to 1 for this analysis). We drew inferences from these models using two-sided Wald tests. We used Spearman’s rank-correlation test to calculate other correlations between aberrations, touch response, mobility and aggregates. A *P*-value <0.05 was considered statistically significant. Statistics were calculated using Wolfram Mathematica version 9.0.1 (Champaign, IL, USA) or SPSS version 21 (Armonk, NY, USA).

## Figures and Tables

**Table 1:** Summary of significant results of branches (b) and extended outgrowths (o) from RNAi screen

*Gene*	*Class*	*Effect on growth*	*Mean per cell (gene/control)*	*% Change*	P*-value versus control*
*icd-1*	ER stress/UPR	b↑	0.817/0.186	339	0.000
*pas-6*	Ubiquitin pathway	b↑	0.672/0.186	261	0.000
*lgg-1*	Autophagy	b↑	0.589/0.186	217	0.004
*chn-1*	Ubiquitin pathway	o↓	0/0.328	dec	0.000
*lgg-1*	Autophagy	o↓	0.101/0.328	69	0.004
*zig-4*	Cytoarhcitecture	o↓	0/0.328	dec	0.010
*phi-32*	Ub pathway	o↓	0.099/0.328	70	0.011

Abbreviations: b, branches; ER, endoplasmic reticulum; GFP, green fluorescent protein; o, outgrowths; RNAi, RNA interference; UPR, unfolded protein response.

*P*_*mec-4*_GFP animals (strain ZB4064) were treated with RNAi for the corresponding gene starting at the L4 stage and imaged on day 5 of adulthood. Percent change was calculated with respect to empty vector control. In cases where the mean was 0, an overall decrease or increase is reported.

**Table 2:** Summary of significant results of kinks (k) and loops (l) from RNAi screen

*Gene*	*Neuron type*	*Class*	*Effect on growth*	*Mean per cell (gene/control)*	*% Change*	P*-value versus control*
*pek-1*	ALM	ER Stress/UPR Components	k↑	0.264/0	inc	0.000
*pek-1*	PLM	ER Stress/UPR Components	k↑	1.107/0	inc	0.000
*pas-3*	PLM	Ubiquitin Pathway	k↑	0.458/0	inc	0.000
*gob-1*	PLM	ER Stress/UPR Components	k↑	0.192/0	inc	0.009
*chn-1*	ALM	Ubiquitin Pathway	l↑	1.5/0.187	802	0.006
*vha-13*	ALM	Lysosome Function	l↑	0.923/0.187	394	0.035
*cup-5*	PLM	Lysosome Function	l↑	1.509/0.622	143	0.000

Abbreviations: ER, endoplasmic reticulum; GFP, green fluorescent protein; K, kinks; l, loops; RNAi, RNA interference; UPR, unfolded protein response.

*P*_*mec-4*_GFP animals (strain ZB4064) were treated with RNAi for the corresponding gene starting at the L4 stage and imaged on day 5 of adulthood. Percent change was calculated with respect to empty vector control. In cases where the mean was 0, an overall decrease or increase is reported.
