# Supplementary figures and images for: Morphological remodeling of C. elegans neurons during aging is modified by compromised protein homeostasis
Source: NPJ Aging Mech Dis. 2016 Apr 7;2:16001–. doi: 10.1038/npjamd.2016.1 (PMC4920063; doi:10.1038/npjamd.2016.1)

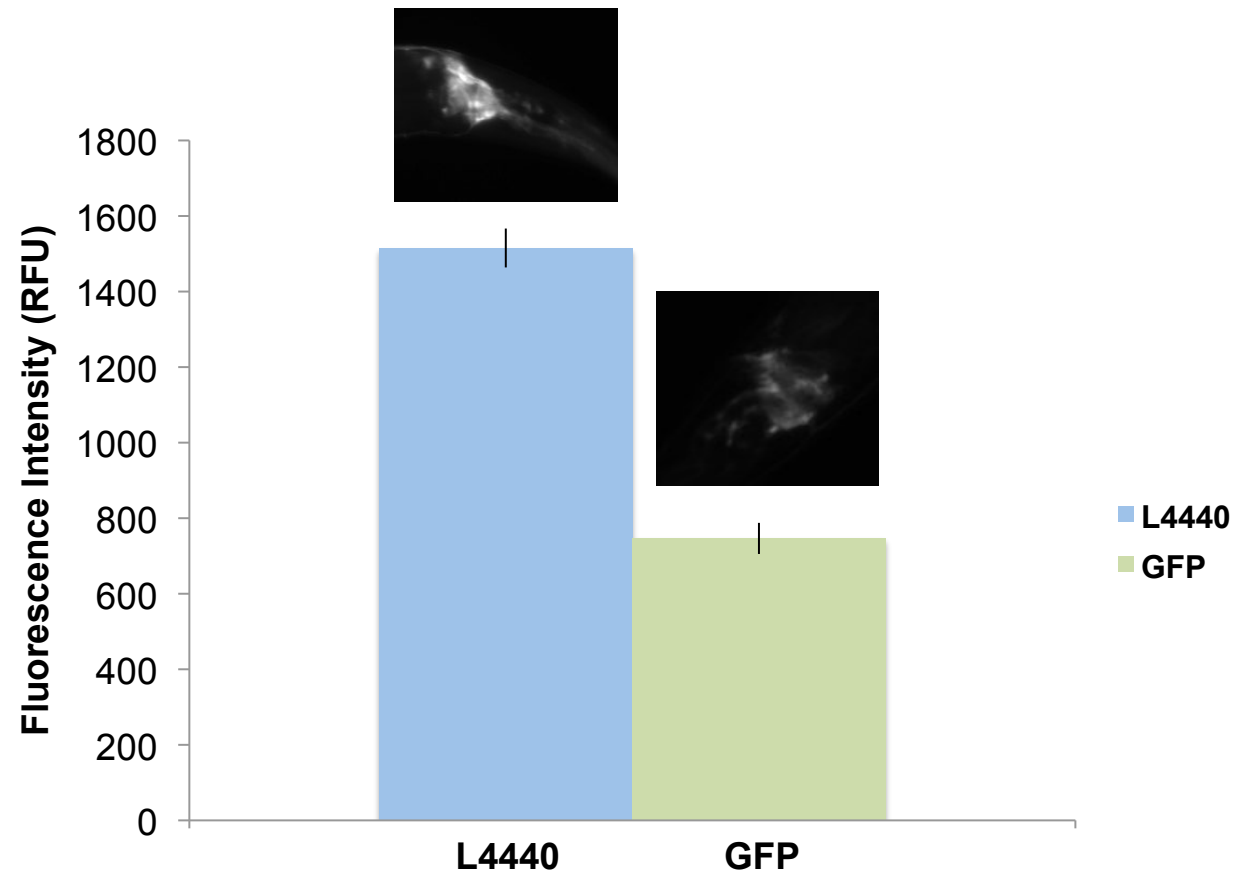

Supplement: Supplementary Figure 1 [file npjamd20161-s1.pdf]

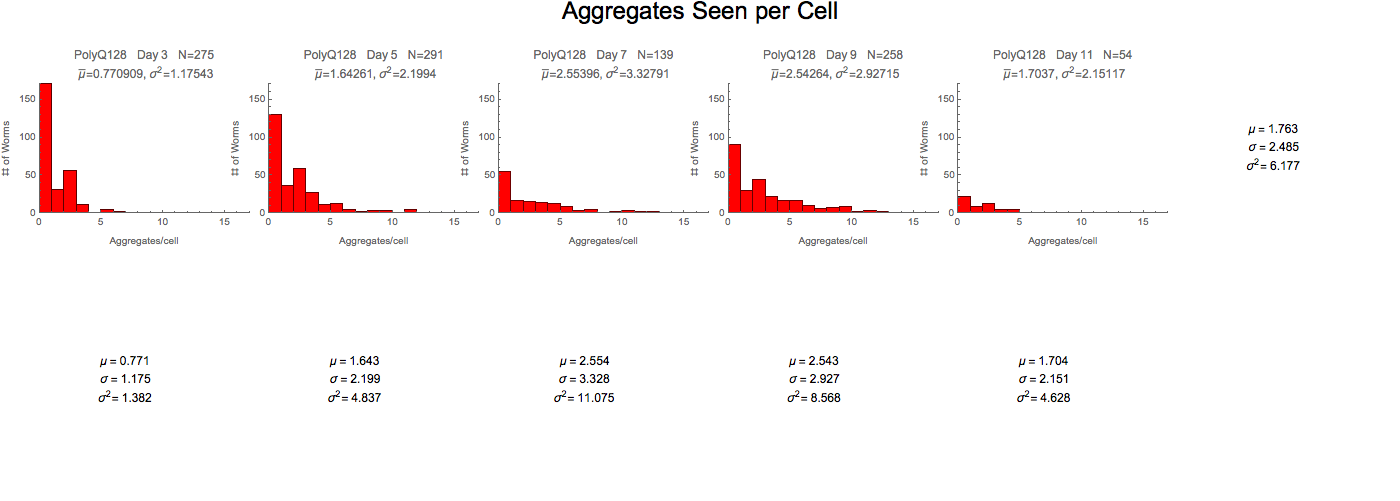

Supplement: Supplementary Figure 2 [file npjamd20161-s2.png]

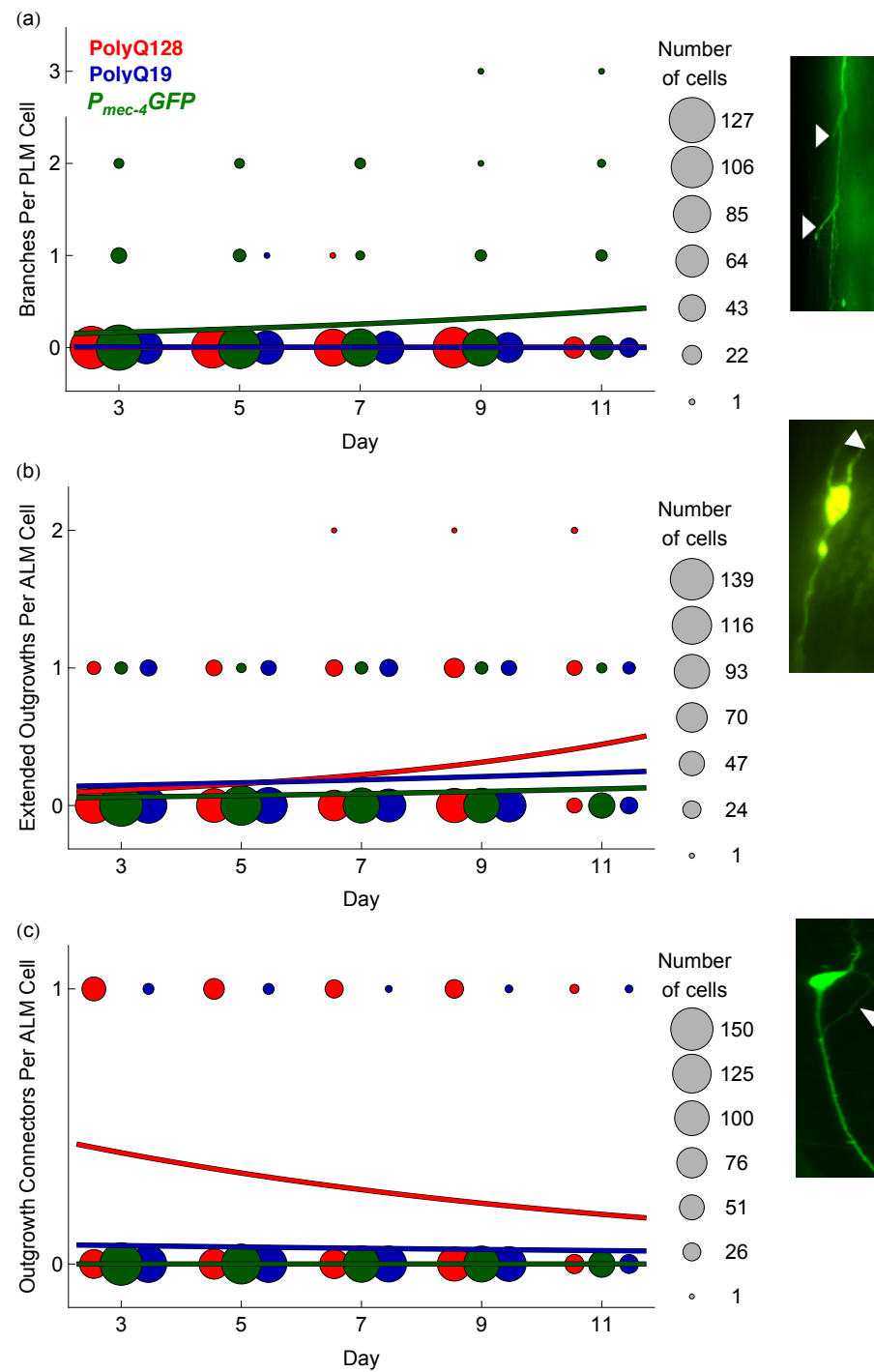

Supplement: Supplementary Figure 3 [file npjamd20161-s3.pdf]
